# The effect of iron on the expression levels of calcium related gene in cisplatin resistant epithelial ovarian cancer cells

**DOI:** 10.37349/etat.2021.00048

**Published:** 2021-08-30

**Authors:** Bahire Kucukkaya, Demet Erdag, Fahri Akbas, Leman Yalcintepe

**Affiliations:** 1 Department of Biophysics, Faculty of Medicine, Istanbul Yeni Yuzyil University, 34010 Istanbul, Turkey; 2 Department of Computer programming, Vocational School, Biruni University, 34010 Istanbul, Turkey; 3 Department of Biophysics, Faculty of Medicine, Bezmialem Vakif University, 34093 Istanbul, Turkey; 4 Department of Biophysics, Istanbul Faculty of Medicine, Istanbul University, 34093 Istanbul, Turkey; Regina Elena Cancer Institute, Italy

**Keywords:** Cisplatin, drug resistance, calcium, iron, inositol 1,4,5-triphosphate receptor, ryanodine receptor, sarco/endoplasmic reticulum Ca^2+^ ATPase, Na^+^/Ca^2+^ exchange

## Abstract

**Aim:**

Anticancer drugs (chemotherapeutics) used in cancer treatment (chemotherapy) lead to drug resistance. This study was conducted to investigate the possible effect of iron on calcium homeostasis in epithelial ovarian cancer cells (MDAH-2774) and cisplatin-resistant cells of the same cell line (MDAH-2774/DDP).

**Methods:**

To develop MDAH-2774/DDP cells, MDAH-2774 (MDAH) cells were treated with cisplatin in dose increases of 5 μM between 0 μM and 70 μM. The effect of iron on the viability of MDAH and MDAH/DDP cells was determined by 3-(4,5-dimethylthiazol-2-yl)-2,5-diphenyltetrazolium bromide test at the end of 24 h incubation.

**Results:**

At increasing iron concentrations in MDAH and MDAH/DDP cells, the mRNA gene of fifteen genes [inositol 1,4,5-triphosphate receptor *(IP_3_R)1/2/3*, ryanodine receptor *(RYR)1/2*, sarco/endoplasmic reticulum Ca^2+^ ATPase *(SERCA)1/2/3*, Na^+^/Ca^2+^ exchange *(NCX)1/2/3*, and plasma membrane Ca^2+^ ATPase *(PMCA)1/2/3/4*] associated with Ca^2+^ differences in expression were determined by quantitative reverse transcription-polymerase chain reaction. Changes in *IP_3_R2, RYR1, SERCA2, NCX3, PMCA1,* and *PMCA3* gene expressions were observed in iron treatment of MDAH/DDP cells, while changes were detected in iron treatment of MDAH cells in *IP_3_R1/2/3, RYR1/2, SERCA1/2/3, NCX2/3,* and *PMCA1* expressions.

**Conclusions:**

This changes in the expression of calcium channels, pumps, and exchange proteins in the epithelial ovarian cancer cell line and in cisplatin-resistant epithelial ovarian cancer cells suggest that iron may have an important role in regulating calcium homeostasis. Due to differences in the expression of genes that play of an important role in the regulation of calcium homeostasis in the effect of iron, drug resistance can be prevented by introducing a new perspective on the use of inhibitors and activators of these genes and thus cytostatic treatment strategies.

## Introduction

Cancer is an important health problem affecting many people around the world. For this reason, great interest and economic effort is spent to discover new strategies and methods for the prevention and treatment of cancers worldwide [1]. Although chemotherapy is the main strategy in cancer treatment, drug resistance to chemotherapeutic drugs is one of the reasons that hinder success. While many cancers initially show sensitivity to chemotherapy, drug resistance appears over time in the effectiveness of various mechanisms. Clinically, drug resistance may be present before treatment (internal) or develop as a result of treatment (acquired) [2]. Deaths of among women worldwide result from ovarian cancer which is one of the ten most prevalent types of cancer. Epithelial tumor cancer account for 75% of all ovarian cancers and is the most common ovarian cancer [3].

Iron and calcium are the essential elements in the fulfillment of vital functions of cells. Calcium is a secondary messenger associated with basic physiological role, such as cell viability, migration, cycle control, apoptosis, and gene expression [4]. Most cellular events are controlled by intracellular calcium ion ([Ca^2+^]_i_). The intracellular Ca^2+^ concentration is kept at very low levels (~10^−7^ mol/L), but the release of a very small amount of Ca^2+^ from the intracellular organelles (~10^−5^ mol/L) or the flow of Ca^2+^ into the cell from outside (~10^−5^ mol/L) the cell can generate important signals by activating the signal transduction cascades in the cell [5]. In general, opening of permeable ion channels to Ca^2+^ increases [Ca^2+^]_i_, while active Ca^2+^ carriers are responsible for returning [Ca^2+^]_i_ to rest. Intracellular Ca^2+^ homeostasis is regulated through diverse calcium channels, pumps and exchange proteins. In non-excitable cells, ER is the main organelles that store Ca^2+^ in the cell, and the channels that give Ca^2+^ from these organelles into the cell; inositol 1,4,5-triphosphate receptors (*IP_3_Rs*) and ryanodine receptors (RYRs) [6]. RYRs are mainly found in cells that can be stimulated, while *IP_3_Rs* are found in cells that cannot be stimulated. So far, 3 types of *IP*_3_ receptors as *IP_3_R1, IP_3_R2* and *IP_3_R3* have been defined [4]. Stimuli that belong to different stress elements (e.g., immobilization stress, oxidative stress, and hypoxia) as well as apoptosis may modulate *IP_3_Rs*. Expression of IP_3_Rs may be increased or decreased relying on the length and strength of the stress stimuli [7].

*RYRs* have three isoforms in mammals as *RYR1*, *RYR2*, and *RYR3*. *RYR1* is mainly found in skeletal muscle, while *RYR2* is found in cardiac muscle. *RYR3* is mostly found in the thalamus, hippocampus, corpus striatum and smooth muscle. In addition, *RYR3* is found in mammalian skeletal muscle cells during development [5]. Another Ca^2+^ pump in the endo/sarcoplasmic reticulum (ER/SR) membrane is sarco/endoplasmic reticulum Ca^2+^ ATPase (*SERCA*). This pump helps to regulate the concentration of cytologic Ca^2+^ by pumping the calcium directly out of the cell or organelles [8, 9]. In addition, plasma membrane Ca^2+^ ATPase (*PMCA*) pump in the eukaryotic cell membranes conduce to the regulation of cytoplasmic Ca^2+^ concentration by removing the excess Ca^2+^ in the cell. Na^+^/Ca^2+^ exchange (NCX) proteins remove Ca^2+^ from the cell to maintain cellular homeostasis. Ion channels were shown to act as the main regulators of specific pathways associated with proliferation, migration, or survival in the progression of cancer and also play a role in different types of cancer such as colon, prostate and breast cancer [4].

Iron is an essential trace element and is the most plenty transition metal in the human body. Because of iron’s ability to receive and donate electrons, it is a crucial component of storage and transport molecules and enzymes involved in energy production and intermediate metabolism when converting between iron (Fe^3+^) and iron (Fe^2+^) oxidation states. Since the ribonucleotide reductase (RR) enzyme, which is responsible for the synthesis of deoxyribonucleotides, is iron-dependent, it is vitalin the process of iron cell division [10]. It has also been shown that iron availability regulates other proteins involved in DNA damage detection by modulating the cell cycle, such as murine double minute 2 (Mdm2), growth arrest and DNA damage (GADD45) and p21/WAF1. In addition, iron is the functional component of proteins containing heme and iron-sulfur cluster synthesized in mitochondria. Therefore, iron is an important element for survival, growth, and differentiation of cell [10]. Furthermore, changes are expected in iron metabolism in the tumor cells, which generally have rapid growth rates. Iron homeostasis is very well regulated in the metabolism [2]. The excess or deficiency of iron causes formation of reactive oxygen species (ROS) in the organism and the organism is damaged [2]. The formation of ROS and especially reactive hydroxyl radicals through Fenton reaction causes mutations and severe cellular damage. As a result of these reactions, studies on iron metabolism in cancer cells have shown that tumor cells require higher iron concentrations, and iron uptake protein genes have been shown to be highly expressed [2]. However, the studies are limited on general iron metabolism in drug-resistant tumor cells. In this research, we examined the effect of iron on the gene expression of major channels (*IP3R1/2/3*, *RYR1*, and *RYR2*), pumps (*SERCA1/2/3*, *PMCA1/2/3*, and *PMCA4*), and exchangers (*NCX1/2*, and *NCX3*) involved in the calcium regulation process in ovarian cancer cells and cisplatin resistant ovarian cancer cells. Iron was noted to indicate differential effects on the studied genes with a concentration-independent activity. To cope with drug resistance, defining the modelling Ca^2+^ channels/transporters/exchangers can supply a promising chemotherapy for cancer resistance.

In this study, epithelial ovarian cancer (EOC) cells and the same cell line resistant to cisplatin, fifteen calcium-associated genes (*IP_3_R1/2/3*, *RYR1/2*, *SERCA1/2/3*, *NCX1/2/3*, and *PMCA1/2/3/4*) expressions were examined using the quantitative reverse transcription-polymerase chain reaction (RT-PCR) method.

## Materials and methods

### Cell line and chemicals

Human EOC cell line, MDAH cells were obtained from the Department of Biophysics in Istanbul Faculty of Medicine and maintained in Dulbecco’s modified Eagles medium (DMEM) F-12 supplemented with 10% heat-inactivated fetal calf serum (FCS), and 100 IU/mL penicillin-streptomycin. Incubation conditions at 37°C in a humidified atmosphere of 5% CO_2_ were maintained in a Heraeus incubator (Hanau, Germany). Cisplatin (Sigma) was applied to the MDAH cell line with dose increments. The subline resistant to cisplatin (MDAH/DDP) from parental MDAH cell line was developed by increasing the 5 µM dose, stepwise, 5 µM to 70 µΜ for cisplatin. Before the dose increments, adapted cells became confluent in respective drug concentrations and maintained at least for three weeks (approximately 3–4 weeks). Cells selected in 25 µΜ cisplatin were maintained in medium with 25 µΜ cisplatin for assays [11].

### 3-(4,5-dimethylthiazol-2-yl)-2,5-diphenyltetrazolium bromide assay

Cell viability was evaluated using 3-(4,5-dimethylthiazol-2-yl)-2,5-diphenyltetrazolium bromide (MTT) cell staining [12]. MDAH and MDAH/DDP groups were added to 96-well plates in triplicate in each well and 2 × 10^5^ cells were incubated at 37°C for 24 h. In DMEM containing 10% FCS, the MDAH/DDP group was prepared using various iron concentrations (from 0 μM to 80 μM) for 24 h at 37°C. MTT solution was added to the cells that was treated with iron. The experiment was incubated at 37°C for 30 min and 4 h. Dimethyl sulfoxide (DMSO, 100 µL) was added to each well after removal of medium. Absorbance values were measured at 570 nm wavelength and the relative cell viability (%) was expressed as a percentage relative to the untreated control cells.

### RNA isolation and real-time quantitative RT-PCR

For isolation of total RNA, MDAH and MDAH/DDP cells were grown in four wells for each process, and total RNA was isolated using the total RNA extraction kit (Thermo). cDNA was synthesized from RNA (500 ng) using the Revert Aid First Strand cDNA synthesis kit (Thermo Scientific), according to the manufacturer’s instructions. RT-PCR was performed using *IP_3_R1/2/3*, *RYR1/2*, *SERCA1/2/3*, *NCX1/2/3* and *PMCA1/2/3* and *PMCA4*-specific primers (Table 1). The original PCR primers were designed using primer 3 software (http://primer3plus.com/). Primer sequences of genes are shown below (Table 1). Quantitative assays were tested with SYBR Green Master Mix (Thermo). The reaction mixture was prepared in a total volume of 20 µL. PCR conditions were performed at 95°C for 3 min, then 40 cycles at 95°C for 10 s and 60°C for 30 s. The target mRNA amount in the samples was calculated by normalizing according to the actin β-mRNA level. Each reaction was performed three times and mean values were determined. The transcriptional gene expression, which was later called gene expression, was changed to ethanol-treated controls as fold change (FC) and was defined by the ΔΔCt method [13].

**Table 1.** Primary sequences of genes

**Common name**	**Primary couples (5’–3’)**	**Product size (bp)**
*IP_3_RI*	Forward: TGACGAGAACCTGCCCTAT Reverse: TCCTTTCGCCATCTTGCT	304
*IP_3_R2*	Forward: GCAATCGTGTCTGTTCCA Reverse: TCTTCAAGTCTCAGCATCG	332
*IP_3_R3*	Forward: GCCTACTATGAGAACCACACG Reverse: CAGAAGAGCAATGAGATGAGAG	389
*RYRI*	Forward: TGACTACCATCAGCACGACA Reverse: ACGAAGACGGCAGGAAATA	297
*RYR2*	Forward: AACAAGTACATGCCTGGTTTGC Reverse: TTGTTCTCATCAGGGAACAGGG	348
*SERCAI*	Forward: GTGATCCGCCAGCTAATG Reverse: CGAATGTCAGGTCCGTCT	361
*SERCA2*	Forward: CGCTACCTCATCTCGTCCA Reverse: TCGGGTATGGGGATTCAA	406
*SERCA3*	Forward: GATGGAGTGAACGACGCA Reverse: CCAGGTATCGGAAGAAGAG	409
*NCX1*	Forward: GTCGCACTTGGAACATCA Reverse: CCAGGGAGGAGAAGAAAA	373
*NCX2*	Forward: CGGTGGATAAACTCATCAAGAA Reverse: CAGGGCAACGAAGACAACA	359
*NCX3*	Forward: GAGATGGGAAAGCCAGTAT Reverse: ATGCCACGAAAACAACAG	430
*PMCA1*	Forward: GAGATGGGAAAGCCAGTAT Reverse: ATGCCACGAAAACAACAG	418
*PMCA2*	Forward: GAGATGGGAAAGCCAGTAT Reverse: ATGCCACGAAAACAACAG	214
*PMCA3*	Forward: TGGTCCTCTACTTTGTGATTG Reverse: TGGTGGTATAGGCACTGTTG	417
*PMCA4*	Forward: CTGTGCGTAATGAAGTGC Reverse: AGTCCCGGTAAGCTATG	279

### Statistical analysis

Data were given as mean with standard deviation; at least 3 replicates were performed for each experimental set up. The statistical significance between control condition and each of the exposure samples were obtained with Student’s *t*-test. Data were expressed as mean ± standard error of the mean (SEM).

## Results

### Iron treatment of cells

**Figure 1.**
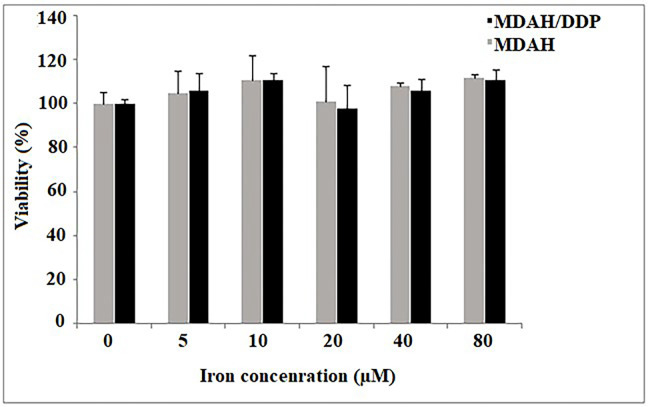
The viability of MDAH and MDAH/DDP cells after treatment with iron for 24 h was determined by performing the MTT test as described in the Materials and Methods section. Values were results from three independent experiments. Student’s “*t*-test” was done

MDAH and MDAH/DDP cells were incubated with iron for 24 h at increasing concentrations of iron. Iron was statistically determined to have no effect on the viability of MDAH and MDAH/DDP cells with the MTT test (Figure 1).

### Expression of Ca^2+^ related genes

**Figure 2.**
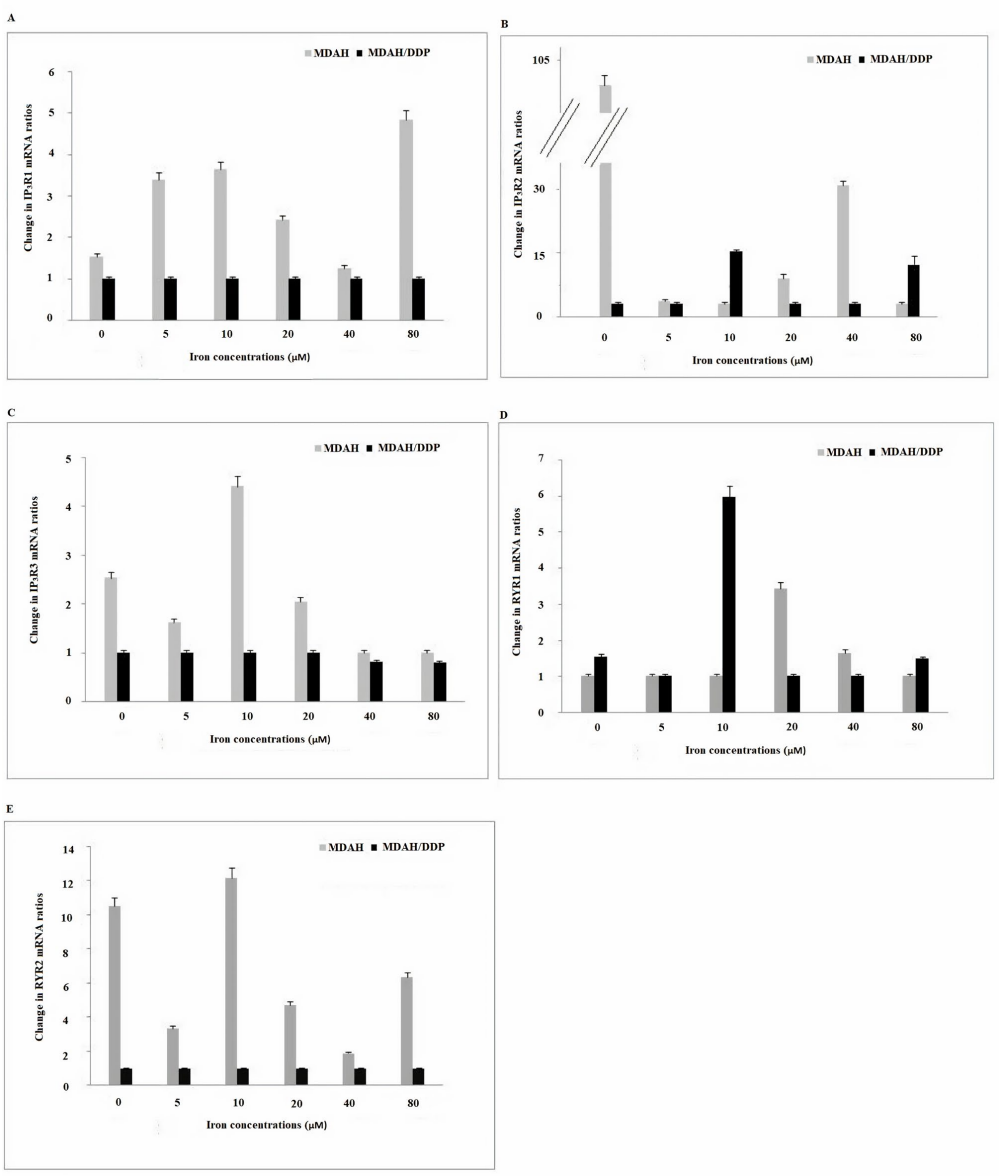
Gene expression of the *IP3R1/2/3*, *RYR1* and *RYR2* Ca^2+^ channels in the ER in MDAH and MDAH/DDP cells treated with increasing iron concentrations. The levels of change in mRNA gene expression rates for each sample were determined with respect to actin by RT-PCR method. A: *IP3R1*; B: *IP3R2*; C: *IP3R3*; D: *RYR1*; E: *RYR2*

Differences in mRNA expression of fifteen genes (*IP_3_R1/2/3*, *RYR1/2*, *SERCA1/2/3*, *NCX1/2/3*, and *PMCA1/2/3/4*) associated with Ca^2+^ were analyzed by quantitative RT-PCR in increasing iron concentrations in MDAH and MDAH/DDP cells. 2-ΔΔCt values that were calculated from the measurement of Ca^2+^-related mRNA expressions from MDAH and MDAH/DDP cells were compared. A two-fold gene expression difference (*P* < 0.05) was taken as the cut-off level. At increasing iron concentrations in MDAH and MDAH/DDP cells, mRNA expression levels of *IP_3_R1/2/3*, *RYR1* and *RYR2* Ca^2+^ channels in the ER and releasing Ca^2+^ out of the cell were compared (Figure 2).

While no change in *IP_3_R1* gene expression was observed in increasing concentrations of iron in MDAH/ DDP cells, a significant rise in *IP_3_R1* gene expression was observed in the treatment of MDAH cells with 5 µM, 10 µM and 80 µM iron. While an increase in *IP_3_R2* gene expression was determined in the treatment of MDAH/DDP cells with 10 μM and 80 μM iron, a significant reduction in iron expression and *IP_3_R2* gene expression was determined in MDAH cells. In addition, *IP_3_R2* mRNA level was seen to be significantly higher in MDAH cells than other *IP_3_R1* mRNA and *IP_3_R3* mRNA expression levels. There is no change in *IP_3_R3* gene expression determined in increasing concentrations of iron in MDAH/DDP cells, but an increase in *IP_3_R3* gene expression was determined in the treatment of MDAH cells with 5, 10 and 20 µM iron (Figure 2).

While an increase in *RYR1* gene expression was observed at 10 µM iron concentration of MDAH/DDP cells in iron treatment of MDAH/DDP and MDAH cells, a significant increase in 20 µM iron treatment of MDAH cells and later decrease in both cell groups were detected. On the other hand, no change was observed in *RYR2* gene expression in iron treatment of MDAH/DDP cells, while a decrease in *RYR2* mRNA expression in MDAH cells at 5 µM, 20 µM and 40 µM was observed. In addition, the examination of the gene expression levels of *RYRs* in MDAH and MDAH/DDP cells, showed that the level of *RYR2* mRNA expression was quite lower in MDAH/DDP cells compared to expression in MDAH cells.

**Figure 3.**
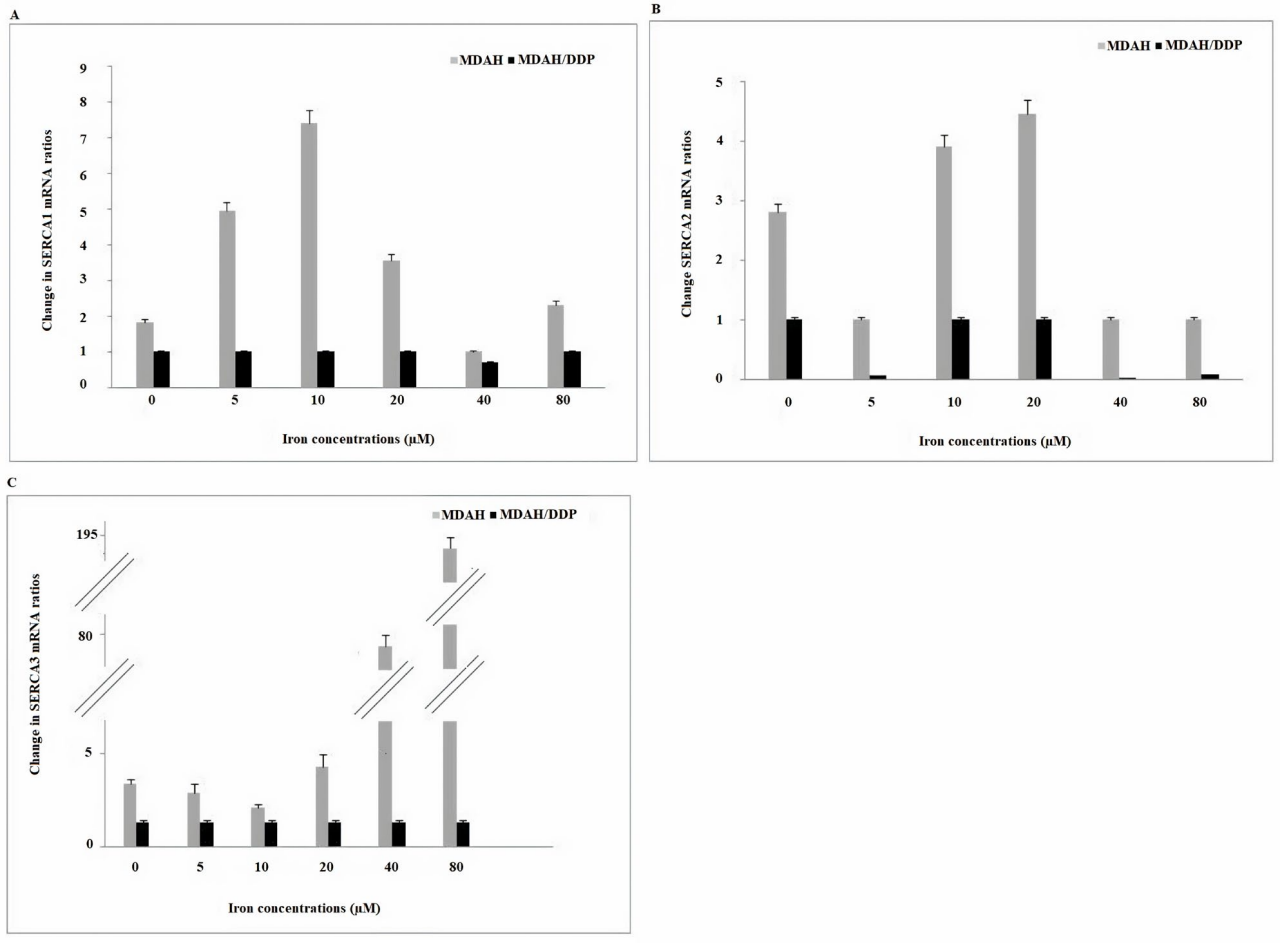
Gene expression of *SERCA1/2* and *SERCA3* Ca^2+^ pumps in the ER at increasing iron concentrations in MDAH and MDAH/DDP cells. The levels of change in mRNA expression rates for each sample were determined with respect to actin by RT-PCR method. A: *SERCA1*; B: *SERCA2*; C: *SERCA3*

Thapsigargin sensitive *SERCAs* are responsible for the conservation and regeneration of agonist sensitive internal stores. No change in *SERCA1* mRNA gene expression was observed in the treatment of MDAH/DDP cells with increasing concentrations of iron, while an increase in MDAH cells was determined at 10 µM (Figure 3). As a result of the treatment of MDAH/DDP and MDAH cells with increasing iron concentrations, a decrease in *SERCA2* gene expression was determined in the treatment of MDAH/DDP cells with 5, 40 and 80 μM iron. However, when *SERCA2* mRNA gene expression was examined in non-iron treated MDAH and MDAH/DDP cells, it was found lower in MDAH/DDP cells. In addition, a significant rise in *SERCA3* gene expression was determined in MDAH cells in increasing iron concentrations, while no change was detected in MDAH/DDP cells.

**Figure 4.**
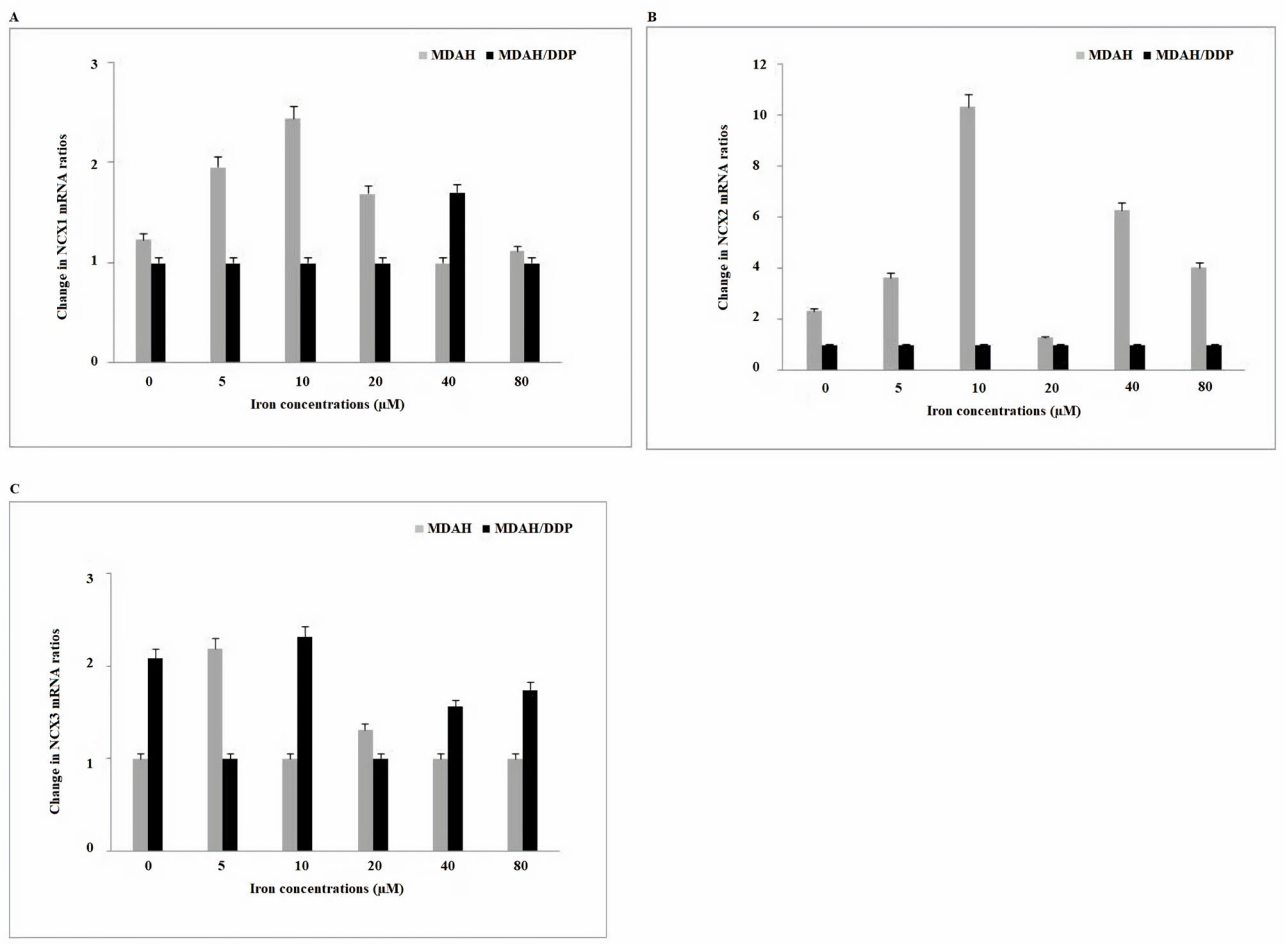
Gene expressions of *NCX1/2* and *NCX3* Ca^2+^ exchange carriers found in plasma and nuclear membrane at increasing iron concentrations in MDAH and MDAH/DDP cells. The levels of change in mRNA expression rates for each sample were determined with respect to β-actin by RT-PCR method. A: *NCX1*; B: *NCX2*; C: *NCX3*

*NCX* proteins send Ca^2+^ into cytosol or organelle using the electrochemical gradient of Na^+^. When *NCX1* mRNA expressions in increasing iron concentrations of MDAH/DDP and MDAH cells were examined, no significant change was observed in both cell groups (Figure 4). While no change in *NCX2* gene expression was observed in increasing concentrations of iron of MDAH/DDP cells, an increase in *NCX2* mRNA expression in MDAH cells was determined in the treatment of MDAH cells with 10 μM, 40 μM and 80 μM iron. In the treatment of MDAH/DDP cells with 5μM and 20 μM iron, a decrease in *NCX3* gene expression was detected, while an increase in *NCX3* mRNA gene expression was determined in the treatment of MDAH cells with 5 μM iron.

**Figure 5.**
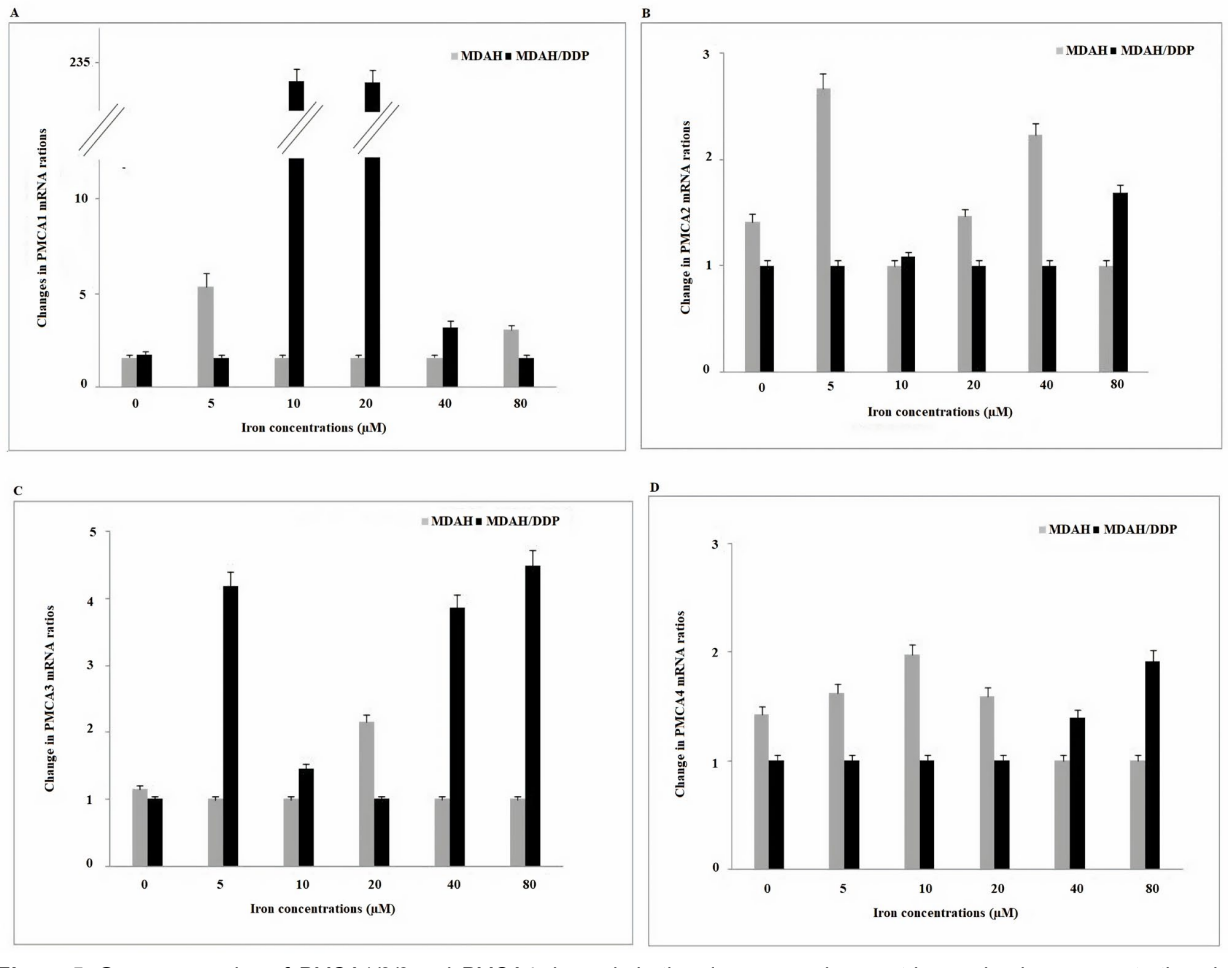
Gene expression of *PMCA1/2/3* and *PMCA4* channels in the plasma membrane at increasing iron concentrations in MDAH and MDAH/DDP cells. The levels of change in mRNA gene expression rates for each sample were determined with respect to P-actin by RT-PCR method. A: *PMCA1*; B: *PMCA2*; C: *PMCA3*; D: *PMCA4*

*PMCA* is the Ca^2+^ channel protein responsible for keeping the intracellular critical Ca^2+^ concentration low. A significant increase in *PMCA1* gene expression was detected in the treatment of MDAH/DDP cells with 10 μM and 20 μM iron (Figure 5). However, an increase in *PMCA1* gene expression was detected in the treatment of MDAH cells with 5 μM and 80 μM iron. An increase in *PMCA3* expression was determined in the treatment of MDAH/DDP cells with 5 μM, 40 μM and 80 μM iron, while no significant change was detected in the expression of *PMCA3* in the treatment of MDAH cells with iron. No change in *PMCA4* gene expression levels was observed in increasing iron concentrations of MDAH/DDP and MDAH cells. No change in *PMCA2* mRNA gene expression was observed when MDAH and MDAH/DDP cells were treated with iron.

## Discussion

Cisplatin is one of the most effective anticancer agents known as cisplatinum or *cis*-diamminedichloroplatin (II). It has been widely used for the treatment of different types of neoplasms, including head, neck, lung, ovarian, breast, brain, kidney and testicular cancers, and leukemia. In general, cisplatin and other platinum-based compounds are considered cytotoxic drugs that inhibit DNA synthesis and mitosis, killing cancer cells and causing apoptotic cell death. It is characterized by the effect of various molecular mechanisms, production of ROS and lipid peroxidation, stimulation of p53 signaling, and cell cycle arrest, down regulation of proto-oncogenes, anti-apoptotic proteins, and oxidative stress in both internal and external pathways in apoptosis [14]. However, cisplatin chemotherapy also causes important side effects such as hepatotoxic, nephrotoxic, cardiotoxic, neurotoxic and/or hematotoxic damage. In addition, cisplatin resistance may develop, and cancer may relapse in the treatment of some patients with cisplatin [15]. Therefore, cisplatin resistance is a major barrier to successful treatment outcomes and limits its clinical application. Although cisplatin and its derivatives cause various side effects and resistance development in ovarian cancer, they are used in basic therapy. Cisplatin is used with other chemical agents or compounds to overcome barriers to the treatment of ovarian cancer with cisplatin. Therefore, it is very important to explain the resistance mechanism, and the mechanism of action of cisplatin is unknown. In this study, MDAH/DDP cells were treated with increasing iron concentrations to investigate the effect of iron on calcium channels, carriers, and exchange proteins of mRNA gene expressions in ovarian cells in which ovarian cancers developed cisplatin resistance, and 15 genes (*IP_3_R1/2/3*, *RYR1/2*, *SERCA1/2/3*, *NCX1/2/3*, *PMCA1/2/3/4*) related to Ca^2+^ were examined by quantitative RT-PCR method and compared with MDAH cells.

The ER acts as the main Ca^2+^ storage in the cell. It is also the organelle that regulates ER cell apoptosis, survival, and ATP production. Intracellular Ca^2+^ levels are controlled by Ca^2+^ release from ER and Ca^2+^ entry from outside the cell. When calcium arrived the cytoplasm, Ca^2+^ often occurs complexes with calmodulin to regulate a diversity of kinases and cyclins, which regulate cell proliferation and apoptosis [16, 17]. Ca^2+^ is a regulater for global cellular processes in such a way that any disturbances to Ca^2+^ homeostasis via changings in expression of Ca^2+^ channels and Ca^2+^ binding proteins can disrupt the cell-cycle [18]. Ca^2+^ levels of cytosolic the activity of guanosine exchange factor (GEF), a Ras stimulator, and GTPase activating protein (GAP), a Ras inhibitor. When GEF is activated, Ras stimulates the proliferative mitogen-activated protein kinase (MAPK) pathway. This results in upregulation of cyclin D1 in the cytoplasm and phosphorylation of RB1 and then, release of the E2F transcription factor which initiates the cells transition into S-phase. This relationship between calcium and RB1 show that elevated cytosolic Ca^2+^ levels can regulate activation of the MAPK pathway, causing removal of the G1/S transition. The first stage in metastasis is the loss of cell-cell connections. Focal adhesion kinase (FAK) is a protein tyrosine kinase that increases turnover of cell-cell contacts [19]. FAK is overexpressed in a number of tumors and is a significant indicator of patient survival. Rised intracellular Ca^2+^ upregulates FAK at focal adhesions through phosphorylation by the calmodulin-dependent protein kinase II (CaMKII) [20]. Thus, abnormal signaling resulting in increased intracellular Ca^2+^ levels can lead to a raise in FAK and a higher turnover rate for cell-cell attachments [21].

Under normal conditions, intracellular Ca^2+^ stimulates cell survival; however, increased Ca^2+^ levels under the influence of stimuli such as cisplatin and thapsigargin may trigger apoptosis. The release of Ca^2+^ from ER into the cell takes place by the channels of two different classes, *IP_3_Rs* and *RYRs* [15]. Luminal ER Ca^2+^ levels are protected with *SERCA* (*SERCA1/2/3*) calcium pumps [22]. The activation of phospholipase C-coupled receptors (some G-protein coupled receptors) causes IP_3_ formation and then the release of Ca^2+^ from calcium-sensitive channels (*IP_3_R1*, *IP_3_R2*, *IP_3_R3*) in the ER membrane [23]. The cytosolic calcium signal induced by *IP*_3_ is highly dependent on the expression profile of *IP_3_R* and the sensitivity of *IP_3_R* subtypes to *IP*_3_ is also different [24]. In addition, *IP_3_R* regulates cellular functions such as secretion, motion, and autophagy [23]. Gene expression of *IP_3_R* has been shown to decrease in cancer and especially *IP_3_R3* isoform in glioblastoma, gastric, small, and non-small lung, and colorectal cancer [25]. In addition, *IP_3_R* may play a role in cancer progression and metastasis, but its mechanism of action has not been revealed [25]. In our study, no change in *IP_3_R1* and *IP_3_R3* mRNA gene expression was observed in iron treatment of MDAH/DDP cells. However, an increase in the *IP_3_R1* mRNA level was observed in iron treatment of MDAH cells, while a decrease in the *IP_3_R3* mRNA level was determined. An increase in *IP_3_R2* gene expression was determined in iron treatment of MDAH/DDP cells while a significant reduction in iron treatment of MDAH cells was determined. In addition, the comparison of MDAH/DDP cells and MDAH cells without iron conditions showed that *IP_3_R2* expression was 100 times higher in MDAH cells. These findings suggest that *IP_3_R2* may have an important role in MDAH/ DDP and MDAH cells in Ca^2+^ homeostasis influenced by iron.

While *RYRs*, a class of Ca^2+^ releasing channels, are rarely associated with cancer, they were reported to regulate proliferation of melanocytes and migration of T cells and astrocytes [25, 26]. *RYR1* is mainly expressed in skeletal muscle and although in small amounts, it is found in the heart muscle, smooth muscle, stomach, kidney, thymus, cerebellum, Purkinje cells, adrenal gland, testicle in the ovaries and B-lymphocytes [27]. *RYR2* is mostly found in the heart muscle, while in small amounts, it is expressed in Purkinje cells, stomach, kidney, adrenal gland, ovary, thymus, and lung. *RYR3* is expressed in hippocampal neurons, thalamus, Purkinje cells, corpus striatum, skeletal muscle, coronary smooth muscle cells, lung, kidney, ileum, jejunum. High conductivity *RYR* ion channels can form rapid transient increases of cytosolic Ca^2+^. Analysis of the primary structure of *RYRs* reveals various functional motifs seen in other proteins; however, the role of these motifs in the function of *RYR* has not been clarified yet [28]. In this study, an increase in gene expression levels of *RYR1* was determined in iron treatment of MDAH and MDAH/DDP cells. In MDAH/DDP cells, there was no change in the effect of iron in the level of *RYR2* mRNA, whereas in MDAH cells, the effect of iron was decreased. While differences in *RYR2* mRNA gene expression levels are observed in MDAH cells, the absence of a significant change in MDAH/DDP cells indicates that *RYR2* is not affected by iron concentrations. However, significant increase in *RYR1* expression and subsequent reduction in increasing concentrations of iron in resistant MDAH cells showed that *RYR1* can contribute to regulation of Ca^2+^ levels in resistant MDAH cells.

The *SERCA* pump is from the family of P-type ATPases and functions in intracellular calcium signal and homeostasis. *SERCA* has 3 isoforms. *SERCA1/2* is expressed in some tissues, while *SERCA3* is ubiquitous. Change in *SERCA* expression has been recorded in oral squamous cancer, choroid plexus, thyroid, lung, colon, acute promyelocytic leukemia, cervical and breast cancers [29–34]. During tumorigenesis in colon cancer, *SERCA3* gene expression is gradually decreasing, while becoming almost zero in poorly differentiated tumors. Similarly, *SERCA3* gene has not been expressed when compared to normal forms such as ductal carcinomas, acute promyelocytic leukemia cells and Burkitt lymphoma cells [33]. When *SERCA1/2/3* mRNA gene expressions in iron effect were examined in cisplatin resistant MDAH/DDP cells, there was no change in *SERCA1* and *SERCA3* mRNA expressions, whereas *SERCA2* mRNA gene expression was decreased. In iron treatment of MDAH cells, an increase in *SERCA1/3* mRNA levels was determined, while the effect of iron was decreased in the level of *SERCA2* mRNA. Changes in *SERCA2* mRNA levels in cisplatin resistant cells under the influence of iron suggest that there may be a contribution to *SERCA2* in the regulation of Ca^2+^ homeostasis in these cells. It also shows that changes in *SERCA1/2/3* mRNA levels in MDAH cells under the influence of iron may play a role in regulating Ca^2+^ homeostasis in ovarian cancer epithelial cells.

There are 3 different isoforms of NCX proteins, NCX1, 2, and 3, that allow Ca^2+^ to be excreted from the plasma membrane. Pelzl et al. [35] showed that mRNA levels of *NCX3* in cisplatin resistant ovarian cells increased in relation to the sensitive cell line. Moreover, as a result of pre-incubation of cells with an *NCX* inhibitor KB-R7943, the inhibitor showed a blunted effect on [Ca^2+^]_i_, and significantly augmented the cisplatin-induced cell death of cisplatin resistant ovary carcinoma cells without effecting cisplatin-induced cell death of cisplatin sensitive ovary carcinoma cells. In another study by Pelzl et al. [36], therapy sensitive D283, and therapy resistant UW228-3 medulloblastoma cells were used and they found that *NCX3* silencing enhanced the apoptosis. In our study, we also found that *NCX3* gene expression levels were increased in MDAH/DDP cells at presence of iron. Our results support to previous studies and it is thought that *NCX3* plays an important role in the drug resistance mechanism.

*PMCA* is expressed ubiquitously, and has critical role for maintaining low resting ([Ca^2+^]i) in all eukaryotic cells. Cytotoxic Ca^2+^ overload has such a central role in cell death and the *PMCA* takes a role for the delicate balance between cell survival, and cell death. In general, the decreased *PMCA* expression leads to cytotoxic Ca^2+^ overload and Ca^2+^ dependent cell death both in apoptosis and necrosis. Therefore, *PMCA* is suggested to have a paradoxical role in cell survival in drug resistance. Our results also showed that the upregulation of *PMCA1* at 10 µM, and 20 µM iron and *PMCA3* at 5 µM, 40 µM and 80 µM in MDAH/DDP cells. Upregulation of *PMCA1* and *PMCA3* mRNA levels in cisplatin resistant EOCs under the influence of iron may suggest that *PMCAs* may be a separate pathway for resistant cells to escape from cell death and/or, perhaps a new route through which they efflux drugs out. In addition, some isoforms of *PMCA* in colorectal and breast cancers have been shown to have significant changes in gene expression [37–42] and colorectal tumor tissues decreased *PMCA4* compared to normal colon cancers.

Barabas and Faulk [43] have shown that doxorubicin-resistant human chronic myelogenous leukemia cells (K562) and promyelocytic leukemia cells (HL60) have up-regulated transferrin receptor (TfR) expression. They have also found that the number of transferrin receptors have been down-regulated by calcium channel blockers, such as verapamil, which is a well-known P-glycoprotein inhibitor and a calcium channel blocker. Doxorubicin-resistant HL60 cells did not overexpress P-glycoprotein, in comparison to drug-sensitive cells, by down regulating TfR. Davies et al. [44] have determined that TfR can be upregulated by growth hormones and this induction has been demonstrated to be triggered by calcium channels [45] and the blockade of such mechanism could re-sensitize drug-resistant cells [46]. This may explain the association of TfR with drug resistance through the function of calcium-sensitive receptors [37]. Finally, we may suggest in accordance with our results that there is a relation between calcium homeostasis and iron uptake and that this response is different in cisplatin-resistant cells than in cisplatin-sensitive cells.

In conclusion, changes were observed in expression of *IP_3_R1*, *IP_3_R2*, *IP_3_R3*, *RYR1*, *RYR2*, *SERCA1*, and *SERCA3* genes in iron treatment of MDAH cells, while changes were determined in *IP_3_R2*, *RYR1*, *SERCA3*, *PMCA1*, and *PMCA2* gene expression in iron treatment of MDAH/DDP cells. These results suggest that changes in the expression of calcium channels, pumps and exchange proteins in the EOC cell line and cisplatin-resistant EOC cells may have an important role in iron regulation of calcium homeostasis. Due to the differences in gene expressions involved in regulation of calcium levels under the effect of iron, drug resistance can be prevented by introducing a new perspective on the use of inhibitors and activators of these genes and thus cytostatic treatment strategies.

## Abbreviations

EOC: epithelial ovarian cancer
FAK: focal adhesion kinase
*IP_3_Rs*: inositol 1,4,5-triphosphate receptors
MTT: 3-(4,5-dimethylthiazol-2-yl)-2,5-diphenyltetrazolium bromide
NCX: Na^+^/Ca^2+^ exchange
PMCA: plasma membrane Ca^2+^ ATPase
ROS: reactive oxygen species
RT-PCR: reverse transcription-polymerase chain reaction
RYRs: ryanodine receptors
SERCA: sarco/endoplasmic reticulum Ca^2+^ ATPase
TfR: transferrin receptor

## Declarations

### Copyright

© The Author(s) 2021.
